# HER2-low and tumor infiltrating lymphocytes in triple-negative breast cancer: Are they connected?

**DOI:** 10.1186/s13058-024-01783-z

**Published:** 2024-03-11

**Authors:** Ximena Baez-Navarro, Nadine S. van den Ende, Anh H. Nguyen, Renata Sinke, Pieter Westenend, Johannes Bastiaan van Brakel, Claudia Stobbe, Johan Westerga, Carolien H. M. van Deurzen

**Affiliations:** 1 Department of Pathology, Erasmus University Medical Center, 3015 GD Rotterdam, The Netherlands; 2 Department of Pathology, HMC, The Hague, The Netherlands; 3 Department of Pathology, Pathan B.V., Franciscus Gasthuis & Vlietland, Rotterdam, The Netherlands; 4 Laboratory of Pathology, PAL Dordrecht, Dordrecht, The Netherlands; 5 Department of Pathology, Skåne University Hospital, Malmö, Sweden

**Keywords:** Triple-negative breast cancer, HER2-low, TILs, Neoadjuvant chemotherapy

## Abstract

Most patients with triple-negative breast cancer (TNBC) are not candidates for targeted therapy, leaving chemotherapy as the primary treatment option. Recently, immunotherapy has demonstrated promising results in TNBC, due to its immunogenicity. In addition, a novel antibody–drug conjugate, namely, trastuzumab-deruxtecan, has shown effectiveness in TNBC patients with low-HER2 expression (HER2-low). These novel treatment options raise the question about the potential association between the density of stromal tumor-infiltrating lymphocytes (sTILs) and the level of HER2 expression. We aimed to evaluate the association between the level of HER2 expression (HER2-low versus HER2-0) and density of sTILs in TNBC patients, and how they impact the response to neoadjuvant chemotherapy (NAC). This was a retrospective multicenter study including all TNBC patients diagnosed between 2018 and 2022. Central pathology review included sTILs percentages and level of HER2 expression. Tumors were reclassified as either HER2-0 (HER2 IHC 0) or HER2-low (IHC 1 + or 2 + with negative reflex test). Various clinicopathologic characteristics, including sTILs density, and response to NAC were compared between HER2-0 and HER2-low cases. In total, 753 TNBC patients were included in this study, of which 292 patients received NAC. Interobserver agreement between the original pathology report and central review was moderate (77% had the same IHC status after reclassification in either HER2-0 or HER2-low; *k* = 0.45). HER2-low TNBC represented about one third (36%) of the tumors. No significant difference in sTILs density or complete pathologic response rate was found between HER2-0 and HER2-low cases (*p* = 0.476 and *p* = 0.339, respectively). The density of sTILs (≥ 10% sTILs vs. < 10%) was independently associated with achieving a pCR (*p* = 0.011). In conclusion, no significant association was found between HER2-low status and density of sTILs nor response to NAC. Nonetheless, sTILs could be an independent biomarker for predicting NAC response in TNBC patients.

## Introduction

Female breast cancer (BC) is the most commonly diagnosed cancer worldwide [1]. Triple-negative breast cancer (TNBC) constitutes 12–17% of all BCs and it is characterized by the lack of estrogen receptor (ER), progesterone receptor (PR) and human epidermal growth factor receptor 2 (HER2) expression [2, 3]. Because of this, patients with TNBC are not eligible for endocrine or HER2-targeted therapies, leaving chemotherapy as the therapeutic option of choice in most cases [4]. TNBC is considered an aggressive BC subtype with relatively high recurrence, metastatic, and mortality rates [5].

At present, treatment decisions for TNBC are based on baseline prognostic clinicopathologic characteristics. Several studies reported that immune response biomarkers, including the density of stromal tumor-infiltrating lymphocytes (sTILs), have an important prognostic and predictive role in TNBC [6, 7]. Patients with a higher density of sTILs at diagnosis have a better disease free and overall survival compared to TNBC patients with a low density of sTILs [8–10]. Several studies including TNBC patients who underwent neoadjuvant chemotherapy (NAC) demonstrated that a high density of sTILs was associated with higher rates of complete pathological response (pCR), independent of other clinicopathological prognostic factors or the chemotherapy regimen [6, 11, 12]. Furthermore, a number of clinical trials have reported that the combination of immunotherapy (e.g., pembrolizumab or atezolizumab) and chemotherapy showed higher rates of pCR than chemotherapy alone [13, 14].

A novel therapeutic option that has demonstrated promising results in a subgroup of TNBC patients is the antibody drug conjugate (ADC) called trastuzumab-deruxtecan (T-DXd) [15]. Notably, this drug showed effectiveness in patients with HER2 overexpression (HER2-positive) but also in those with a low level of HER2 expression (HER2-low) [15]. HER2-low BCs has been defined as tumors with a HER2 immunohistochemistry (IHC) score of 1 + or 2 + with a negative molecular reflex test [16, 17]. In a recent phase III clinical trial including 557 patients with HER2-low BC, from which 63 had TNBC, those who had been treated with T-DXd showed longer progression free survival and overall survival compared to the patients treated with regular chemotherapy [15]. This favorable response of HER2-low tumors to T-DXd has opened the question whether these tumors have clinicopathologic differences compared to TNBC tumors with less HER2 expression (HER2 IHC 0), since this could have consequences for novel treatment strategies, i.e., immunotherapy for immunogenic TNBC and T-DXd for HER2-low TNBC. Several studies reported that within TNBC, HER2-low tumors have a poorer pathologic complete response rate after NAC compared to HER2-0 [18–20]. This could be associated with a lower immune response of the former group. In line with this hypothesis, van den Ende et al. reported that a lower density of sTILs was significantly associated with HER2-low tumors in TNBC [21]. However, despite these studies reporting a favorable response to NAC in HER2-0 TNBC, this is still controversial, since a recent meta-analysis found no statistically significant difference in pCR rates between patients with HER2-low and those with HER2-0 TNBC [22]. Literature exploring the differences in pCR and its association to in immunological response in HER2-low and HER2-0 within TNBC is limited [23, 24].

The aim of this study was to assess the difference in sTILs density between HER2-0 and HER2-low TNBC. In addition, we aimed to compare the pathologic response between HER2-0 and HER2-low BC from patients who received NAC.

## Methodology

### Inclusion criteria

A retrospective multi-institutional study was performed including all patients diagnosed with TNBC according to the original pathology report based on the Dutch National guidelines and the American Society of Clinical Oncology/College of American Pathologists Clinical Practice Guideline (ASCO/CAP) [25], between the 1st of January, 2018 until the 31st of December, 2022. Patients above 18 years of age were included. The type of treatment did not influence eligibility for this study, as both patients who received NAC and those who did not were included. Patients with an equivocal HER2 status were excluded.

### Data collection

Tissue slides from all TNBC core biopsies were collected. Demographic and clinicopathologic data from the biopsies and the resection specimens were gathered from the pathology reports of the institutional database LIMS Sympathy Tieto of the Erasmus Medical Center, and Core-UDPS of Pathan B.V, and PAL Laboratory of Pathology Dordrecht.

Several clinical characteristics were collected including sex, age at diagnosis (date of core biopsy diagnosis), and type of performed surgery. Histopathologic features from the core biopsy included histologic subtype (according to the World Health Organization) [26], histologic grade (including number of mitosis), angioinvasion and Ki67 proliferation index. The presence of macro- and micro-metastases in sentinel node and/or an axillary nodal dissection were used to determine the nodal status (positive or negative). If a patient had positive nodal status determined by fine needle aspiration (FNA) before receiving NAC and achieved pCR, nodal status from the pre-NAC FNA was considered for analysis.

Furthermore, data regarding the expression of ER, and PR and HER2 was gathered. Hormone receptor (HR) status was defined as positive if more than 10% of the cancer cells showed nuclear ER or PR staining, according to Dutch Guideline for Breast Cancer Treatment, independent of intensity [27]. Since ASCO/CAP guidelines use a 1% cut-off, we also performed the analyzed according to this cut-off [28]. HER2 status was scored according to international guidelines[25].

Additionally, data regarding pCR (defined as the absence of residual invasive breast cancer on histopathologic evaluation of the resected breast specimen and lymph nodes) was included. An incomplete pathologic response was categorized as < 10% residual tumor, 10–50% residual tumor, > 50% residual tumor or no pathologic response[29].

### Consensus meeting

A consensus meeting with all participating pathologists (*n* = 7) was performed before the start of case scoring. This meeting comprised the presentation of the standardized methodology for evaluating sTILs density in BC according to the recommendations by the International TILs working group[6]. Furthermore, the HER2 IHC criteria of the ASCO/CAP guidelines 2018 were reviewed and some exemplary cases of each IHC score were discussed [25].

### HER2 and sTILs scoring

Needle core biopsy slides were stained with Hematoxylin and Eosin staining and the 4B5 HER-2/neu antibody using an automatic immunostainer (Ventana BenchMark Ultra, Roche, Indianapolis, USA). The slides were digitally scanned with the Nanozoomer 2.0-HT (Hamamatsu Photonics, Shizuoka, Japan) which enables Z-stacking. The scanned slides were uploaded to Slide Score B.V. (version 1.2-2022-05-24T15:37:11 (Netherlands Cancer Institute, Amsterdam, The Netherlands). This program blinded the case numbers and randomized the slides for the participating pathologists, who received a personal code to perform the scoring of the density of sTILs and HER2 IHC. sTILs levels were scored according to the recommendations of the International Workgroup for scoring TILs[6]. HER2 IHC was scored according to the 2018 ASCO/CAP guidelines as IHC 0, 1 +, 2 +, 3 + [25]. If the scanned tissue was considered to have insufficient tumor cells for proper scoring, the case was considered as missing data.

The use of coded leftover patient material is in accordance with the Code of Conduct of the Federation of Medical Scientific Societies in the Netherlands[30].

### Data analysis

The tumors were reclassified as HER2-0 (IHC 0) or HER2-low (IHC 1 + or 2 + with negative reflex test). Descriptive statistics were used to present patient and tumor characteristics. Data was presented as relative frequencies (percentage) for categorical variables or median and interquartile range (IQR) for continuous variables. Interobserver agreement was assessed with Cohen´s kappa coefficient. The cut-off values of sTILs (10%) and Ki67 (40%) were based on recent studies reporting that above these cut-off values, these markers may predict response to NAC [31–37]. The association between the HER2 score and the percentage of sTILs was assessed using the Mann Whitney U test (or Chi square, depending on the coding of the variables). pCR was analyzed according to HER2 status using Chi square test. Finally, a univariate and multivariate logistic regression analysis was performed to evaluate the relation between the clinicopathologic characteristics and the pathologic response. Odds ratios was described with 95% confidence interval. A *p* value < 0.05 was considered statistically significant. The analysis was performed in SPSS (IBM Corp. Released 2021. IBM SPSS Statistics for Windows, Version 28.0. Armonk, New York, USA).

### Ethics approval

This work was approved by the Medical Ethics Committee of the Erasmus MC (MEC-2023-0545). The Medical Ethics Committee of the Erasmus MC approved that the rules laid down in the Medical Research Involving Human Subjects Act do not apply to this work. Consequently, informed consent was not needed. The study was performed in accordance with the Declaration of Helsinki.

## Results

### Patient and tumor characteristics

**Fig. 1:**
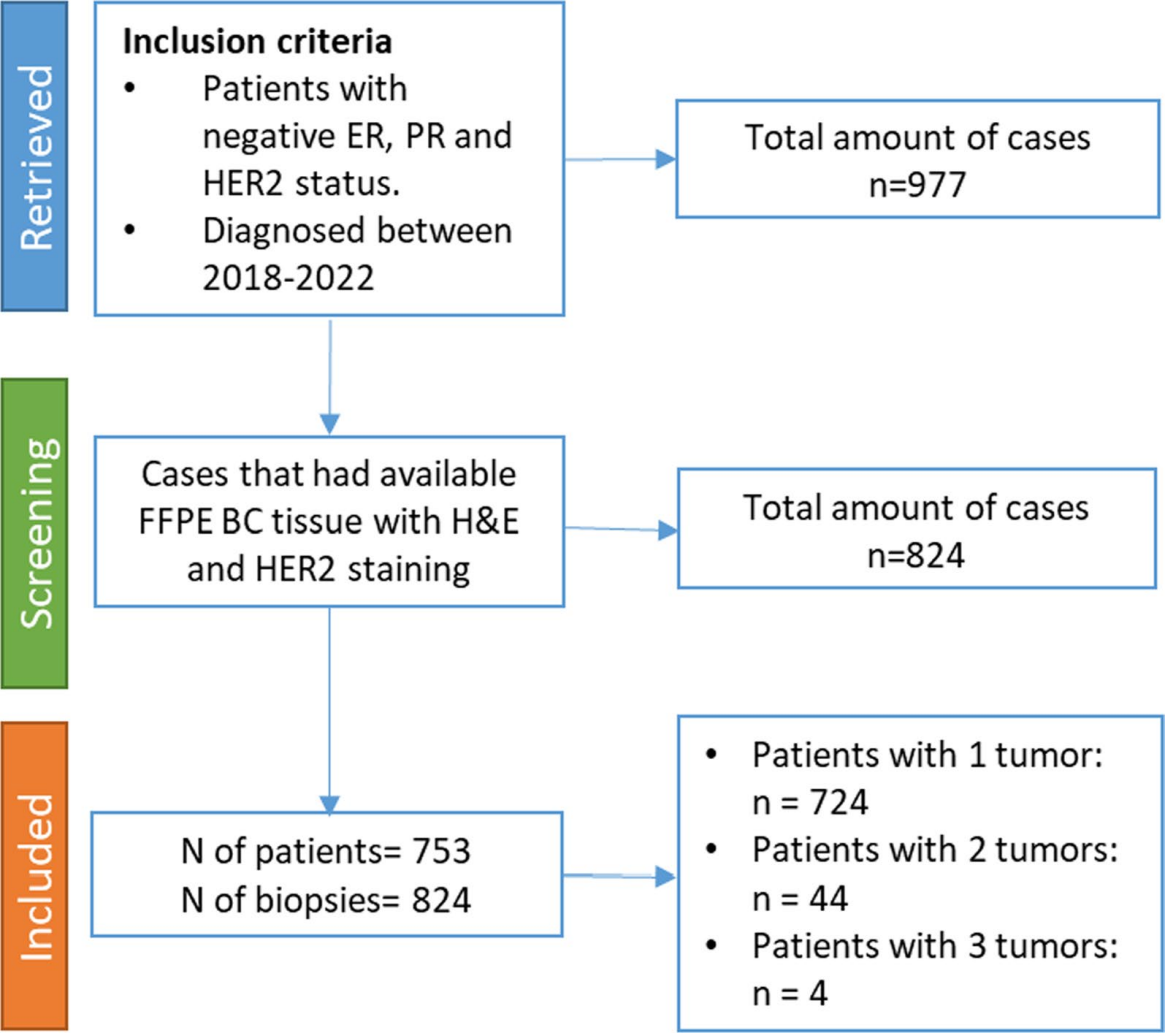
Flow chart of inclusion and exclusion criteria. ER = estrogen receptor; PR = progesterone receptor; HER2 = human epidermal growth factor receptor 2; BC = breast cancer; H&E = hematoxylin & eosin

A total of 977 patients with TNBC were retrieved from the institutional databases from participating medical centers. Among these cases, 753 patients had accessible tumor tissue, enabling analysis of clinicopathologic characteristics. Notably, it was observed that 6% of these patients (*n* = 48) had multiple synchronous tumors, resulting in a total of 824 core biopsies (Fig. 1).

All included patients were women. The median age at diagnosis was 61 (IQR 49–73) years. From the 583 patients that had available surgery data, 56% (*n* = 328) underwent breast conserving surgery while 44% (*n* = 255) underwent mastectomy. The majority of patients (82%, *n* = 677) had a HR expression of < 1%. The remaining cases (11%, *n* = 91) were HR-low (1–9% of positive tumor cells). In 56 patients (7%), the percentage of positive cells was < 10% but the exact percentage was missing. Based on the HER2 IHC score from the original pathology report, 64% (*n* = 527) of the cases had an IHC score of 0, 25% (*n* = 210) of patients had an IHC score of 1 +, and 11% (*n* = 91) of patients had an IHC score of 2 + with negative reflex test. This corresponded to 64% (*n* = 527) of cases with HER2-0 status and 36% (*n* = 294) of cases with HER2-low status. The median density of sTILs was 10% (IQR 5–20%).

### HER2 concordance between multiple tumors

Most of the 48 patients with multiple synchronous tumors (37 out of 48; 77%) had the same IHC score in both tumors. There were 11 cases (23%) in which the tumors had a different IHC score, of which 4 cases had IHC 0 in tumor 1 and IHC 1 + in tumor 2 or vice versa. Six patients had IHC 1 + in tumor 1 and IHC 2 + in tumor 2 or vice versa and 1 patient had IHC 0 in tumor 1 and IHC 2 + in tumor 2. After reclassification of tumors according to the HER2 status (HER2-0 versus HER2 low), 43 of the 48 cases had the same HER2 score in both tumors.

### HER2 central review compared to original pathology report

**Fig. 2:**
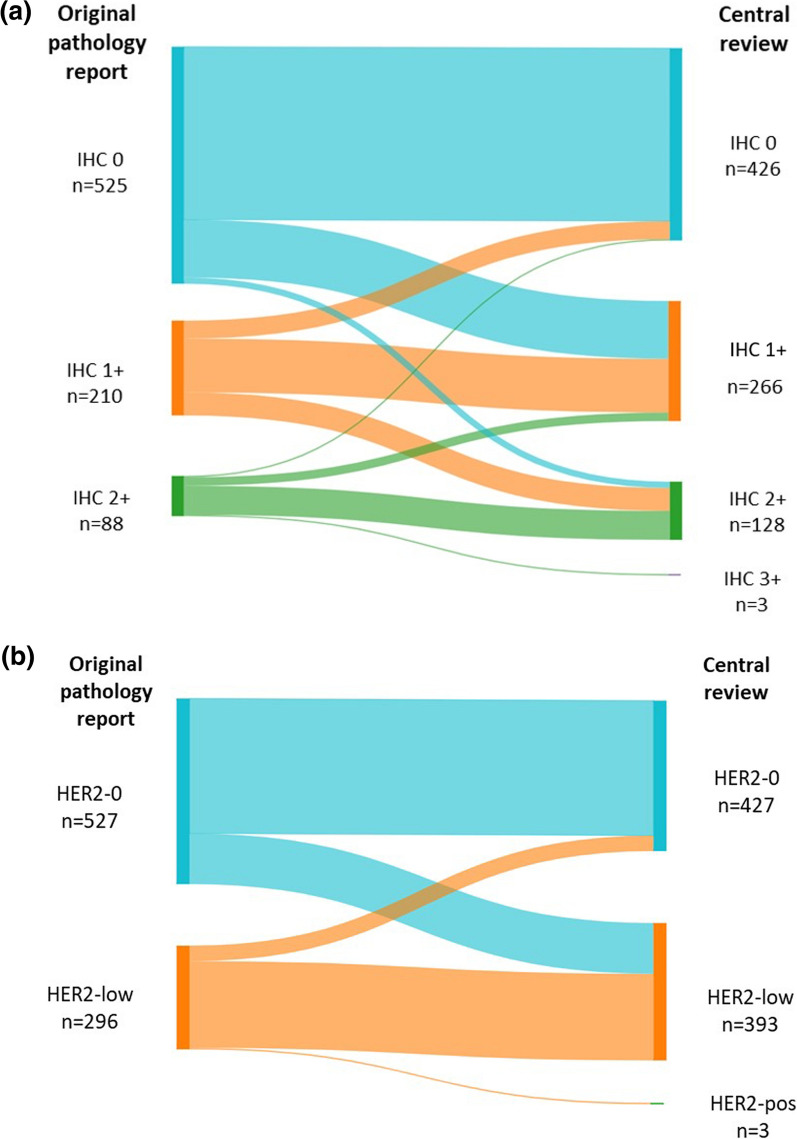
A schematic overview of the immunohistochemistry (IHC) concordance between the original pathology report and the central review result. **A** presents all HER2 IHC scores (0, 1, 2, 3) and **B** presents the IHC classes (HER2-0, HER2-low or HER2-pos)

All given HER2 IHC scores from available biopsies were centrally reviewed and compared to the scores from the original pathology report, which resulted in a moderate interobserver agreement (Cohen´s kappa = 0.45). Figure 2A shows the concordance between both IHC scores. In brief, 823 biopsies were analyzed of which 69% (*n* = 566) had the same IHC score. From the 258 cases that had a different score, 65% (*n* = 168) had a shift from IHC 0 to 1 + or vice versa; 7% (*n* = 17) had a shift of 0 to 2 + or vice versa; and 27% (*n* = 70) had a shift from 1 + to 2 + or vice versa. Notably, there were three cases that were scored as 2 + in the original pathology report that were re-scored as 3 +. These 3 cases had a negative reflex test.

Figure 2B depicts the reclassification of the tumors according to HER2-status (0, low or positive), with a moderate agreement (Cohen’s kappa = 0.52). Around 77% (*n* = 632) of the cases had the same HER2 score. Among the 23% (*n* = 191) of cases that had a different HER2 score, 96% (*n* = 188) changed from HER2-0 to HER2-low or vice versa, and 2% (*n* = 3) changed from HER2-low to HER2-positive.

### Clinicopathologic characteristics according to HER2 status

Table 1 presents the clinicopathologic characteristics from the core biopsy according to HER2 status. Briefly, HER2-low BC was associated with histologic subtype (higher frequency of lobular carcinoma; *p* < 0.001), lower histologic grade (*p* < 0.001), lower Ki67 index (*p* = 0.001), and higher proportion of positive nodal status (*p* < 0.001). There was no difference in density of sTILs between HER2-0 and HER2-low (*p* = 0.571). Subgroup analyses restricted to cases with an HR expression of < 1% did not affect these results.

**Table 1:** Clinicopathologic characteristics according to HER2 status (HER2-0 vs. HER2-low) in TNBC

Tumor characteristics	HER2-0 (*n* = 527)	HER2-low (*n* = 294)	*P* value
*Age (n* = *821)*			
< 50	136 (25.8)	74 (25.2)	0.841
≥ 50	391 (74.2)	220 (74.8)	
Missing	0	0	
*Surgery (n* = *614)*			
Mastectomy	165 (42.4)	112 (49.8)	0.077
Lumpectomy	224 (57.6)	113 (50.2)	
Missing	138	69	
*Histologic type (n* = *818)*			
No special type	474 (90.3)	260 (88.7)	**< 0.001**
Lobular	9 (1.7)	22 (7.5)	
Other	42 (8)	11 (3.8)	
Missing	2	1	
*Grade of invasive component* = *752*			
1	12 (2.5)	21 (7.8)	**< 0.001**
2	207 (42.9)	125 (46.5)	
3	264 (54.7)	123 (45.7)	
Missing	44	25	
*Angioinvasion (n* = *604)*			
Yes	23 (6.1)	17 (7.6)	0.381
Uncertain	4 (1.1)	5 (2.2)	
No	353 (92.9)	202 (90.2)	
Missing	147	70	
*Mitosis (n* = *136)*			
< 8	32 (35.6)	16 (34.8)	0.929
≥ 8	58 (64.4)	30 (65.2)	
Missing	437	248	
*Ki67 (n* = *87)*			
< 40	8 (14.5)	15 (46.9)	**< 0.001**
≥ 40	47 (85.5)	17 (53.1)	
Missing	472	262	
sTILs (median, range)	10 (5–20)	10 (5–26)	0.476
Missing	1	0	
*sTILS (n* = *820)*			
< 10%	238 (45.2)	127 (43.2)	0.571
≥ 10%	288 (54.8)	167 (56.8)	
Missing	1	0	
*Axillary nodal status (n* = *672)*			
Negative	273 (63.9)	123 (50.2)	**< 0.001**
Positive	154 (36.1)	122 (49.8)	
Unknown	100	49	

Bold indicates significance at *P* < 0.05

### Clinicopathologic characteristics according to sTILs

Table 2 presents the association between clinicopathologic characteristics from the core biopsy and a sTIL density of ≥ 10%. In univariate analysis, age < 50 (*p* < 0.001), NST histology (*p* < 0.001), histologic grade 3 (*p* < 0.001), ≥ 8 mitoses mm^2^ (*p* = 0.02), and positive nodal status (*p* = 0.014) were associated with a higher percentage of sTILs. Subgroup analyses restricted to cases with HR expression of < 1% did not affect these results.

**Table 2:** Clinicopathologic characteristics according to sTIL density (< 10% versus ≥ 10%) in TNBC

Tumor characteristics	TILs < 10% (*n* = 368)	TILs ≥ 10% (*n* = 458)	Univariable
			*P* value
*Age (n* = *826)*			
< 50	63 (17.1)	148 (32.3)	**< 0.001**
≥ 50	305 (82.9)	310 (67.7)	
Missing	0	0	
*Histologic type (n* = *822)*			
NST	312 (85.5)	426 (93.2)	** < 0.001**
Lobular	21 (5.8)	10 (2.2)	
Other	32 (8.8)	21 (4.6)	
Missing	3	1	
*Grade of invasive component (n* = *755)*			
1	27 (8.3)	7 (1.6)	**< 0.001**
2	161 (49.4)	171 (39.9)	
3	138 (42.3)	251 (58.5)	
Missing	42	29	
*Angioinvasion (n* = *609)*			
Yes	18 (7)	22 (6.3)	0.727
Uncertain	4 (1.6)	5 (1.4)	
No	236 (91.5)	324 (92.3)	
Missing	110	107	
*Mitosis (n* = *140)*			
< 8	30 (44.8)	19 (26)	**0.02**
≥ 8	37 (55.2)	54 (74)	
Missing	301	405	
*Ki67 (n* = *90)*			
< 40	12 (33.3)	11 (20.4)	0.167
≥ 40	24 (66.7)	43 (79.7)	
Missing	36	54	
*Axillary nodal status (n* = *678)*			
Negative	183 (64.4)	217 (55.1)	**0.014**
Positive	101 (35.6)	177 (44.9)	
Unknown	84	64	
*HER2 status (n* = *820)*			
HER2 0	238 (65.2)	288 (63.3)	0.571
HER2 low	127 (34.8)	167 (36.7)	
Missing	3	3	

Bold indicates significance at *P* < 0.05

### Clinicopathologic features associated with complete pathologic response after NAC

The dataset comprised 292 NAC-treated patients with complete data regarding treatment response in the surgical resection specimen. According to Dutch guidelines for breast cancer treatment, these patients received an antracyclin/taxane-based NAC regimen, supplemented with Carboplatin in patients with stage 3 disease [27].Logistic regression analysis was performed to evaluate the impact of several clinicopathologic characteristics on pCR rate. As presented in Table 3, multivariable analysis showed that having no special type histology (NST) (p = 0.033) and a TIL density of ≥ 10% (*p* = 0.011) were the only factors independently associated with achieving a pCR. Restricting these analysis to patients with an HR expression of < 1% did not affect these results.

**Table 3:** Logistic regression analysis of pathologic characteristics and pCR

	OR	IC 95%	*P* value	*P* value
*Age (n = 292)*				
< 50	Reference	0.430–1.088	0.109	
≥ 50	0.684			
*Histologic type (n* = *291)*				
Lobular or other	Reference	1.111–69.6	**0.039**	**0.033**
No special type	8.792			
*Grade of invasive component (n* = *284)*				
1	Reference			
2	1.439	0.251–8.231	0.683	
3	1.789	0.320–10.01	0.508	
*Angioinvasion (n* = *230)*				
No or uncertain	Reference	0.241–1.677	0.36	
Yes	0.635			
*Mitosis (n* = *95)*				
≥ 8	Reference	0.358–2.263	0.823	
< 8	0.9			
*Ki67 (n* = *27)*				
< 40	Reference	0.056–8.822	0.783	
≥ 40	0.7			
*sTILs percentage (n* = *292)*				
< 10	Reference	1.086–2.899	**0.022**	**0.011**
≥ 10	1.77			
*Axillary nodal status (n* = *260)*				
Positive	Reference	0.483–1.308	0.366	
Negative	0.794			
*HER2 status (n* = *292)*				
HER2 0	Reference	0.498–1.271	0.339	
HER2 low	0.797			

Bold indicates significance at *P* < 0.05

**Fig. 3:**
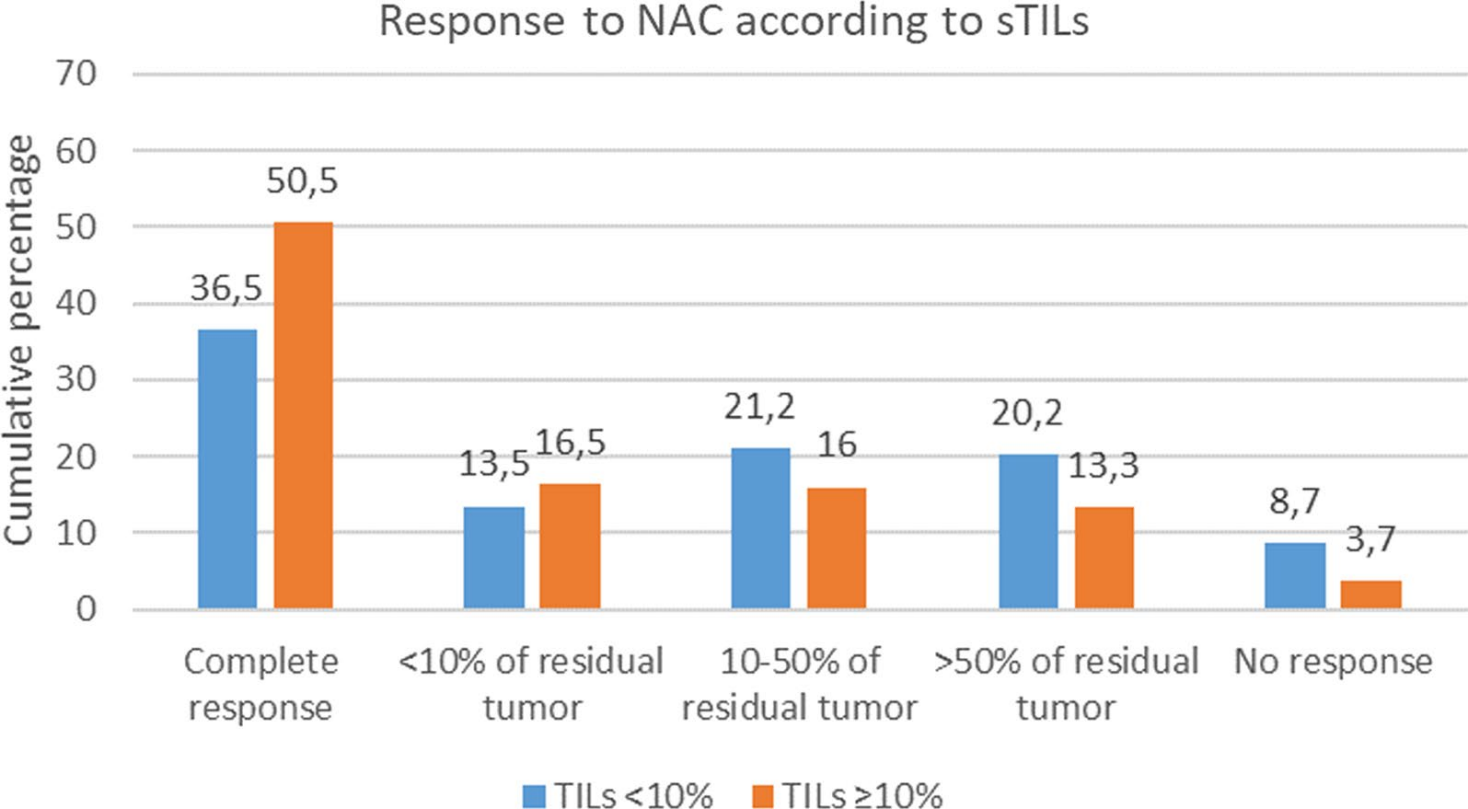
Response to NAC according to density of sTILs. NAC = neoadjuvant chemotherapy; sTILs = stromal tumor-infiltrating lymphocytes

When evaluating the relative differences between sTILs and pCR, it was observed that 50.5% of patients with sTILs ≥ 10% achieved pCR in contrast to 36.5% of those with sTILs < 10% (*p* = 0.021) (Fig. 3). Moreover, we evaluated the variations in the level of treatment response based on the percentage of sTILs. A trend was seen toward a pCR or < 10% residual tumor and a high percentage of sTILs (≥ 10%), whereas the patients with 10% or more residual tumor more often tend to have lower density of sTILs (< 10%) (*p* = 0.054). Based on sTILs, we could not select large subgroups of patients with a very high (e.g., > 90%) or very low pCR rate (e.g., < 10%), although all patients with sTILs ≥ 90% who underwent NAC (*n* = only 3) achieved a pCR.

The response to NAC according to HER2 status was also evaluated. HER2-0 tumors had a higher proportion of patients achieving pCR compared to HER2-low tumors (47.6% vs. 41.9%) but this difference was not statistically significant (*p* = 0.338).

## Discussion

To our knowledge, this is the first comprehensive assessment study aiming to investigate the association between HER2-low status and the immunological response in TNBC. In our analysis, 36% of the TNBC patients had HER2-low status, which is similar to ratios found in other studies [38–40]. HER2-low status was associated with a higher rate of lobular histology, a lower histologic grade, lower Ki67, and a higher nodal positivity rate compared to HER2-0 cases. Similar differences had been reported previously [21, 38, 39, 41]. However, the absolute differences are relatively small, so their clinical relevance remains uncertain.

In our study, no correlation between HER2-low status and density of sTILs was found in TNBC. Literature regarding this topic is very limited [38, 42]. Jacot et al. [42] conducted a retrospective study with 296 patients and reported that HER2-low status had no correlation with sTILs density (5% cut-off value) or PD-L1 expression. A national retrospective study conducted in the Netherlands found a slightly lower median for sTILs level in HER2-low TNBC compared to HER2-0 TNBC, although this discrepancy did not achieve statistical significance[38]. It is important to consider that these studies have certain limitation due to their retrospective design and missing data[38]. In contrast, a recent study from van den Ende et al. reported that lower sTILs density (≤ 10%) was independently associated to HER2-low in TNBC patients and these results were supported by gene expression data[21]. These cases were also centrally reviewed, including sTILs density based on whole tissue slides and HER2 status based on tissue microarrays, all from resection specimens. However, this was a historical cohort (1982 to 2003), which could have affected tissue quality and accurate representation of the current HER2 expression levels in BC patients. In our current study, the assessment of sTILs density was performed on material from the core biopsy. It has been reported that there can be an underestimation of the density of sTILs when scoring on biopsies instead of the resection specimen, and that the discordance in the percentage of sTILs tends to be higher in TNBC cases compare to other BCs [43–45]. Moreover, in the current study it was reported that HER2-0 tumors were associated with the NST histologic subtype and that these tumors were more often grade 3. In addition, high density of sTILs (≥ 10%) was also significantly associated with the NST histologic subtype and grade 3 tumors. Based on this data, one could hypothesize that there is an indirect correlation between HER2 status and density of sTILs, but no direct significant association was found in this study. Further research of the correlation between HER2 status and the density of sTILs in the resection specimens could provide more insight in the potential association between HER2-low status and sTIL density.

As mentioned above, age, NST type, tumor grade 3, ≥ 8 mitosis per mm^2^, and higher positive nodal status were features significantly associated with a high density of sTILs in our cohort, which is in line with literature [8, 46–48]. However, regardless of the consistent association of these clinicopathologic characteristics with a high density of sTILs, their mutual influence and clinical significance in TNBC remains undefined. Moreover, no significant association was found between Ki67 index and density of sTILs, which is in contrast to several previous studies [10, 46, 48–51]. The most likely cause for the deviation in outcomes could be the substantial number of cases with missing data for these parameters in our cohort.

As expected, a significant correlation between achieving pCR and a higher density of sTILs (≥ 10%) was found in this study. Several other studies have reported the robust correlation between sTILs and a higher likelihood of achieving pCR [11, 12, 52–55]. Furthermore, a high density of sTILs has been associated with response to both cytotoxic treatments and immunotherapy, particularly in TNBC [24, 56]. In our study, all patients with sTILs ≥ 90% who underwent NAC achieved pCR, but the absolute number of these patients was very low (*n* = 3).

On the other hand, HER2-low status had no impact in therapy response to NAC within this study. This is in line with a recent meta-analysis including 15 TNBC studies, that found that HER2-low status had no significant impact on pCR rates compared to HER2-0 [22]. Previous studies showed contradictory results regarding the association between HER2-low status and therapy response. In various uni- and multicenter retrospective cohorts, no significant correlation was found between HER2-low status and pCR in the TNBC subgroup [19, 20, 57]. However, in a much larger nation-wide American cohort, a statistically lower pCR rate was associated with the HER2-low status in TNBC [18]. These conflicting outcomes could potentially be influenced by the cohort size, where statistically significant findings are observed only in exceptionally large cohorts [18, 58]. Additionally, a lack of calibration of the IHC assay has been reported for HER2 detection, especially in the context of HER2-low, which raises doubts about the accurate identification of patients with HER2-low and HER2-0 tumors [59]. Overall, these findings indicate that density of sTILs could be a promising independent biomarker for predicting the response to NAC in TNBC patients, while HER2-low status does not appear to have a relevant effect.

The HER2 score variations between the original pathology report and central review were assessed and the interobserver agreement was moderate, with 23% of the cases having a discordant HER2 status (0 vs. low). Comparable interobserver variability using the current diagnostic guidelines has been described before [40, 60, 61]. At the moment, there is not enough evidence to provide a clear recommendation on the optimal classification method for borderline cases between HER2 IHC 0 and IHC 1 +. To address these complex cases, it is advisable to examine them under high power magnification and have them reviewed by a second or third pathologist [16, 17]. It’s crucial to bear in mind that the ASCO/CAP guidelines were established to define HER2 overexpression and its correlation with the response to trastuzumab, rather than to forecast the response likelihood to T-DXd. Furthermore, HER2 status variability was also observed in this cohort when comparing multiple breast tumors. About 10% of patients with multiple tumors displayed a combination of one HER2-0 tumor and one HER2-low tumor. Alongside interobserver variability, tumor heterogeneity within a single tumor and between different tumors could also be a factor for the variation in HER2 status across multiple tumors [62, 63]. When dealing with multiple tumors, clinical guidelines suggest sampling more than one lesion to accurately categorize the disease [27]. Currently, T-DXd is only approved for non-amplified HER2 metastatic BC [64, 65]. However, if this drug becomes part of treatment regimens for early BC in the future, it is important to consider all these factors.

One of the strengths of this study is that it consists of a large and well-documented cohort of patients, as data is collected from multiple centers, enabling a comprehensive representation of patients with TNBC in the Netherlands. Furthermore, to properly estimate the HER2 protein expression levels, central pathology review was performed. However, this study also has some limitations. Due to the retrospective nature of the cohort, some data was missing regarding certain clinicopathologic features, including pre-NAC tumor stage and chemotherapy regimen. For this reason, the findings of some pathologic characteristics should be interpreted with caution. Moreover, the cohort consisted of recently diagnosed patients, limiting the follow-up time, so we couldn’t look at outcome.

## Conclusion

The findings of this study suggest that HER2-low tumors are highly prevalent within TNBC (36%). The HER2-low status is associated with several clinicopathologic characteristics, although the clinical implications are questionable. No significant association was found between HER2-low status and sTILs density nor response to NAC. Nonetheless, the indication that density of sTILs could be an independent biomarker for predicting the response to NAC in TNBC patients was confirmed in this study.

## Data Availability

The datasets used and/or analyzed during the current study are available from the corresponding author upon reasonable request.

## Abbreviations

ASCO/CAP: American Society of Clinical Oncology/College of American Pathologists Clinical Practice Guideline
BC: Breast cancer
pCR: Complete pathologic response
FNA: Fine needle aspiration
HER2: Human epidermal growth factor receptor 2
HER2-low: Low-HER2 expression
IHC: Immunohictochemistry
NAC: Neoadjuvant chemotherapy
NST: No special type
sTILs: Stromal tumor-infiltrating lymphocytes
T-DXd: Trastuzumab deruxtecan
TNBC: Triple-negative breast cancer
